# pH sensitive peptide functionalized nanoparticles for co-delivery of erlotinib and DAPT to restrict the progress of triple negative breast cancer

**DOI:** 10.1080/10717544.2019.1576801

**Published:** 2019-04-08

**Authors:** Xu Wan, Chaoqian Liu, Yinan Lin, Jie Fu, Guohong Lu, Zhengmao Lu

**Affiliations:** a Department of Pharmacy, South Campus, Renji Hospital, School of Medicine, Shanghai Jiaotong University, Shanghai, People's Republic of China;; b Department of General Surgery, Changhai Hospital The Second Military Medical University, Shanghai, People's Republic of China

**Keywords:** Triple negative breast cancer, erlotinib, gamma secretase inhibitor, tumor targeting, nanoaprticles, pH-sensitive

## Abstract

Although a variety of drug delivery strategies have been designed for enhancing the treatment of Triple negative breast cancer (TNBC), combating with TNBCs is still dramatically challenged by the selection of appropriate therapeutic targets and insufficient tumor accumulation or inner penetration of chemotherapeutics. To address these issues, the classical EGFR-inhibitor, erlotinib (EB), was selected as the model drug here and PLA-based nano-platform (NP-EB) was prepared for tumor site drug delivery. Given the significant role of Notch-EGFR interplay in raising severe resistance to EGFR inhibition of EB, gamma secretase inhibitor (GSI)-DAPT was further entrapped into the core of nanoparticles to inhibit the activation of Notch signaling (NP-EB/DART). For achieving the goal of tumor targeting drug delivery, we developed a new peptide CF and decorating it on the surface of EB/DART-dual loaded nanoparticles (CF-NP-EB/DART). Such CF peptide was designed by conjugating two separated peptide CREKA, tumor-homing peptide, and F3, cell penetrating peptide, to together *via* a pH-sensitive hydrazone bond. By this way, the tumor unspecific property of F3 was sealed and significantly reduced the site effects. However, after the nanoparticles reach the tumor site, the pH-sensitive linkage can be broken down by the unique acidic environment of tumor, and subsequently discovered the F3 peptide to penetrate into tumor cells.

## Introduction

TNBCs, belonging to the subtype of epithelial breast tumors, are famous for their negative expression of estrogen receptor (ER), progesterone receptor (PR) and human epidermal growth factor receptor 2 (HER2) (Bauer et al., 2007). As one of the most fatal tumor forms, TNBCs account for 15–20% of all breast cancers and have a featured hallmark of very aggressive behavior (Palma et al., 2015). Although a wide array of treatment strategies and drug delivery approaches have been enhanced recently, treating of TNBCs is still dramatically challenged by the selection of appropriate therapeutic target and the insufficient tumor accumulation and inner penetration of chemotherapeutics.

Epidermal growth factor receptor (EGFR), also known as HER1, is the membrane glycoprotein that belongs to the receptor tyrosine kinases and is encoded by proto-oncogene CerbB-1 (Li et al., 2016; Yan et al., 2017). Under normal physiological conditions, the activated EGFR plays a critical role in the controlling of cell growth, migration and proliferation by transmission of intracellular downstream signaling through a series of signal pathways including Ras/MAPK pathway, PI3K/AKT/mTOR pathway, NF-κB pathway or by the activation of transcription 3 (Baselga & Arteaga, 2005; Crown et al., 2012; Masuda et al., 2012). However, it has been well-defined that the aberrant tyrosine kinase activity by EGFR has a close relation with tumorigenesis in a variety of cancer types such as lung, colon, and breast cancers (Corkery et al., 2009; Efferth, 2012; Quan et al., 2018).

The Notch pathway represents another important mechanism responsible for a broad range of cell fate decisions (Andersson et al., 2011). As a highly conserved developmental pathway, Notch signaling is always activated during normal breast development and the aberrant activation has been demonstrated to be one of the dominant factors that trigger the tumorigenesis of breast cancer (Farnie & Clarke 2007; Robinson et al., 2011). Moreover, there are increasing evidence that hyperactivation of Notch has a favorable effect on the enabling of breast cancer cells to become resistant to a wide range of treatments through a complex mechanism (Baker et al., 2014). Therefore, inactivation of Notch signaling might represent a potential therapeutic approach for enhancing the therapeutic effect of TNBCs.

Studies have demonstrated the superior effect of erlotinib (EB) on EGFR signaling pathway blocking, which results in an obvious inhibition effect of tumor cells growth, invasion and migration (Liu et al., 2017). Additionally, treating with EB also played a promoting role in accelerating cell apoptosis and disruption of angiogenesis through inhibition of the cell cycle protein kinase dependent inhibition protein p27 (p27kip1 protein) (Liao et al., 2014). In the present study, therefore, we have developed PLA-based nano-platforms and used EB as a model drug to treat TNBCs. Given the significant role of Notch-EGFR interplay in raising severe resistance to EGFR inhibition of EB (Arasada et al., 2014), a gamma secretase inhibitor (GSI)-DAPT was further introduced to inhibit the activation of Notch signaling. By utilizing such combination strategy, the growth and progress of TNBCs was hypothesized can be significantly inhibited through a cooperative formation mechanism.

Recognizing the unique advantages of tumor targeting drug delivery (Gao, 2016; Gao, 2017), a tumor homing peptide CREKA was decorated on the surface of EB and DAPT-dual loaded nanoparticles. CREKA is a linear pentapeptide that has been designed to bind to fibrous protein in microenvironment of various types of tumors but not clot protein in normal tissues (Simberg et al., 2007; Song et al., 2014). With regarding to the fact that desirable tumor inner penetration ability composes one of the most necessary attributes for a drug delivery system (Ruan et al., 2017), we further modified the nanoparticles with a cell penetrating peptide F3. F3 peptide is a molecule capable of undergoing an effective cell surface to nucleus transport through binding to nucleolin, a shuttle protein that shows a high expression on a variety of tumor cells and traffics between cell membrane and nucleus (Porkka et al., 2002). However, the F3 peptide is tumor unspecific and may result in serious side effect by enhancing accumulation of nanoparticles in normal tissues. In this case, we conjugated the CREKA molecule with F3 through the acidic sensitive linker-hydrazone. By this way, the cell penetrating ability of F3 was dramatically sealed under normal physic condition and significantly reduced cytotoxicity of drugs to normal tissues. After the nanoparticles reach the tumor site, the pH-sensitive linkage can be broken down by the unique acidic environment of tumor, and subsequently recover the cell penetrating ability of F3 peptide to penetrate into tumor cells. Based on this, we achieved the goal of tumor site specific drug delivery.

## Materials and methods

### Materials and cell culture

We obtained the methoxy poly (ethylene glycol) _3000_-poly (lactic acid) _34000_ (MPEG-PLA) and carboxyl-poly (ethylene glycol) _3400_-poly (lactic acid)_34000_ (HOOC-PEG-PLA) from LACTEL Absorbable Polymers (USA). 1, 10-Dioctadecyl-3,3, 3-tetramethylindo-tricarbocyanineiodide (DiR) was purchased from Biotium (Invitrogen, USA). 3-(4,5-dimethyl-2-thiazolyl)-2,5-diphenyltetra-zoplium bromide (MTT) and coumarin-6 were purchased from Beyotime (Haimen, China). RMPI 1640 medium, fetal bovine serum (FBS) and trypsin-EDTA solutions were obtained from Gibco (CA, USA). All peptides were synthesized by Shanghai Mocell Biotech Co., Ltd (China). Sequences of each peptide used here was CREKA-NH_2_ for C peptide, CKDEPQRRSARLSAKPAPPKPEPKPKKAPAKK-NH_2_ for F3 peptide, and CREKA-CN_2_-CKDEPQRRSARLSAKPAPPKPEPKPKKAPAKK-NH_2_ for CF peptide. All other reagents were of analytical grade and were from Sinopharm Chemical Reagent Co., Ltd (Shanghai, China).

The MDA-MB-231 cell line was obtained from the Chinese Academy of Sciences Cell Bank. Female nude Balb/c mice (18–20 g) were purchased from Shanghai Sino-British Sippr/BK Lab Animal Ltd. (Shanghai, China) and raised under the standard condition. All the animal experiments were conducted in accordance with the guidelines of the Second Military Medical University.

## Establishment of tumor-bearing mice models

The orthotropic breast cancers-bearing mice model were established through inoculating MDA-MB-231 cells into the mammary fat pad of female mice as previously reported (Xiao et al., 2017). Briefly, the MDA-MB-231 cells were trypsinized with 0.25% EDTA and then collect the cell pellets by centrifugation at 1000 rpm for 4 min. After that, MDA-MB-231 cells were resuspended with fresh RMPI 1640 medium and diluted to 2 × 10^4^ cells per 100 μL. Subsequently, 100 μL of the cell suspensions were subcutaneously injected into one of the fourth pair of mammary fat pads of the mice and all the tumor-bearing mice were raised under standard condition for further use.

### Fabrication of nanoparticles

The nanoparticles co-entrapped with EB and DAPT were developed using a previously reported emulsion/solvent evaporation method with a slight modification (Feng et al., 2015). In brief, a blend of MPEG-PLA (18 mg) and HOOC-PEG-PLA (2 mg) were dissolved by 1 ml of dichloromethane. Then 2 mL of 0.6% sodium cholate aqueous solution was added and subjected to sonication (240 W, 5 s for 15 times) in ice bath through a probe sonicator (Scientz Biotechnology Co. Ltd., China). After the dichloromethane was removed by the ZX-98 rotary evaporator (Shanghai Institute of Organic Chemistry, China), the developed nanoparticles (NP-EB/DART) were obtained by centrifugation (14000 rpm, 45 min) under 4 °C. For the preparation of peptide functionalized nanoparticles, the collected nanoparticles were resuspended by distilled water and poured into a penicillin bottle. Then the conjugation of CF peptides on the surface of NP-EB/DART was realized by the amidation reaction under the condition of EDC/NHS (Feng et al., 2015). In brief, EDC (400 mM) and NHS (100 mM) were added into the NP-EB/DART contained penicillin bottle followed by co-incubation for 1 h. After that, nanoparticles were collected again by ultracentrifugation at 14,000 rpm for 45 min. Subsequently, the activated NP-EB/DART was resuspended again and reacted with various peptides under room temperature for 6 h. The molar ratio of peptide to HOOC-PEG-PLA was 1.5:1. CF peptides functionalized nanoparticles were finally obtained by centrifugation (14,000 rpm for 45 min). Drug-, coumarin-6 (C6)- and DiR-loaded NPs were developed by the same method except that 300 µg of drugs, coumarin-6 or 200 µg of DiR was added into the blend in advance.

### Nanoparticles characterization

To observe the morphology of nanoparticles prepared above, a transmission emission microscopy (TEM) (H-600, Hitachi, Japan) was introduced to determine the appearances after negative staining with 1% uranyl acetate. Then a Malvern Nano ZS (Malvern Instruments, UK) was applied to examine the particle size and zeta potential of nanoparticles. The conjugation of peptide on the surface of NP was further confirmed by X-ray photoelectron spectroscopy (XPS) analysis on a RBD upgraded PHI-5000C ESCA system (Perkin Elmer). Additionally, the encapsulation efficiency (EE) and loading capacity (LC) of drugs in NP-EB/DAPT and CF-NP-EB/DAPT were determined by high performance liquid chromatography (HPLC) and calculated as indicated below (*n = 3*).

$$\mathrm{LC}\;\left(\%\right)=\frac{\text{Amount of EB or DAPT in nanopaticles}}{\text{Weight of nanoparticles}}\;\times 100\%$$

$$\mathrm{EE}\;\left(\%\right)=\frac{\text{Amount of EB or DAPT in nanopaticles}}{\text{Total amount of EB or DAPT added}}\;\times 100\%$$

Stability of the developed CF-NP-EB/DAPT was further investigated. For experiments, 5 mg nanoparticles were diluted with 2 mL of saline containing 10% rat plasma and then preserved under room temperature for two weeks. During the experimental periods, particle size of CF-NP-EB/DAPT was determined every two days. For comparison, the NP-EB/DAPT was also subjected to the stability evaluation using the same method.

### *In vitro* drug release studies

Release behaviors of EB and DAPT from the NP-EB/DAPT and peptides modified ones were determined using the dialysis method (*n = 3*). The experiments were conducted in the medium of PBS (pH 6.0 or 7.4) containing 10% rat plasma (with pH 7.4 representing the physiologic pH and pH 6.0 representing the condition of tumor microenvironment). In brief, 1 mL of free drugs or nanoparticle solutions (NP-EB/DAPT, and CF-NP-EB/DAPT) with an equal drug (EB or DAPT) concentration (100 μg/mL) were sealed into a dialysis bag (MWCO = 800), respectively, and immediately placed into 30 mL of release medium. Then the experiment was carried out on the shaking bath for 72 h under the condition of 100 rpm and 37 °C. At each predetermined time point, 100 μL of the medium was withdrawn with an equal volume of fresh release medium was supplemented immediately. The drug concentration of each sample was determined through HPLC analysis.

### Intracellular uptake assay

MDA-MB-231 cells and HUVEC cells were seeded on a 24-well plate with a density of 1 × 10^4^ cells per well. On the second day, the cell culture medium in each well was replaced with 1 mL of fresh medium containing NP-C6, CREKA-NP-C6, F3-NP-C6, or CF-NP-C6 with the coumarin-6 concentration set at 100 μg/mL. After incubation of the cells with different nanoparticles for 1 h, the medium containing unattached nanoparticles were withdrawn and cells were washed three times with cold PBS followed by fixation with 4% paraformaldehyde for 10 min. Finally, the qualitative analysis of cellular uptake of nanoparticles was performed under a fluorescence microscope (Olympus BX 53, Center Valley, PA). For quantitative analysis, tumor cells were processed as above. After the unattached nanoparticles in each well was completely rinsed using cold PBS, the cells were trypsinized with trypsin-EDTA solution and centrifuged at 1000 g for 5 min to collected cell pellets. Finally, the quantitative analysis was conducted through the flow cytometry (FCM) analysis. Of great importance, the cellular uptake assay was also performed on HUVEC cells using the same approaches as above to evaluate the affinity of different nanoparticles to tumor angiogenesis.

### Cytotoxicity of nanoparticles to tumor cells

To evaluate the cytotoxicity of different nanoparticles against tumor cells, MDA-MB-231 cells cultured in different pH conditions were transplanted into 96-well plates at a cell density of 5 × 10^3^ cells per well. After an overnight incubation, the old growth medium in each well was removed and replaced with an equivalent volume of fresh culture medium which contains various nanoparticle formulations, including NP-EB/DAPT, CREKA-NP-EB/DAPT, F3-NP-EB/DAPT, and CF-NP-EB/DAPT. The concentration of nanoparticles was set at 100 μg/mL and the cells treated with drug-free medium were used as control. After the tumor cells were incubated with nanoparticles for 48 h, the drug contained medium was completely withdraw and supplemented with fresh FBS-free medium. Then 20 μL of MTT (5 mg/mL) solution was added into each well and incubated with cells for 4 h. To dissolve the formed formazan crystals, 150 μL of DMSO was added. Subsequently, the absorbance of each well was measured at 570/630 nm using a microplate reader and cell viability was calculated by comparing the absorbance with the untreated cells. In addition, to verify the superiority of combination therapy of EB and DAPT, cytotoxicity of NP-EB, NP-DAPT and NP-EB/DAPT to MDA-MB-231 cells were further determined by MTT assay.

### Migration assay

To investigate the migration ability of breast cancer cells *in vitro*, MDA-MB-231 cells were seeded on the upper transwell chamber and treated by different nanoparticles, including NP-EB/DAPT, CREKA-NP-EB/DAPT, F3-NP-EB/DAPT, and CF-NP-EB/DAPT. The drug concentrations of EB was set at 100 μg/mL. In the meanwhile, the lower chamber was only filled with culture medium containing 10% FBS. After 24 h of incubation, the medium in the lower chamber was removed and the lower surface of the insert was fixed with 4% paraformaldehyde. Thereafter, 0.1% crystal violet was added and allowed to incubate with cells for 15 min. Then the number of migrated cells was observed under a light microscope (Zeiss) and subjected to quantitative analysis.

### Biodistribution of nanoparticles

To investigate the distribution of different nanoparticles *in vivo*, six breast cancer-bearing mice were randomly grouped (*n = 3*) and *i.v.* injected with NP-C6, CREKA-NP-DiR, F3-NP-DiR and CF-NP-DiR, respectively. At 12 h post of the injection, all mice were euthanized with the tumors and major organs including heart, kidneys, liver, spleen, and lung were collected. For quantitative analysis of the drug concentrations in various tissue samples, the obtained tumors and organs were subjected to homogenate followed by determination using the LC/MS/MS.

### *In vivo* antitumor activity

Thirty tumor-bearing mice were randomly divided into five groups (*n = 6*) and treated with various nanoparticle formulations, including NP-EB/DAPT, CREKA-NP-EB/DAPT, F3-NP-EB/DAPT, and CF-NP-EB/DAPT. The drug dosage of EB was set at 5 mg/kg. The mice received the treatment of PBS was used as the control and the therapy was done for three times in a week. Then the anti-tumor effect of different nanoparticles was evaluated by determining the tumor volume using a vernier caliper every two days. The tumor volume was calculated by the following equation: tumor volume = (length × width^2^)/2. Additionally, the weigh change of each mice in different groups was also monitored every two days to reveal the the adverse effect of the formulations.

### Western blot

The effect of EB and DAPT in regulating the expressions of EGFR and Notch signaling pathway in breast cancer cells and tumor tissues were measured by western-blot assay. After the total proteins were exacted from cells and tumor tissues using RIPA buffer, the BCA protein assay kit (Thermo Scientific) was applied to determine the protein concentrations. Then the samples were separated by loading on SDS-PAGE gels (4 15%) and run at 150 V for 80 min. After proteins were transferred to the polyvinylidene difluoride (PVDF) membranes using a wet transfer method at 220 mA and run for 90 min, 5% BSA in a Tris-–buffered Tween 20 solution was added to pre-block the samples for 1 h. Subsequently, primary antibodies including anti-EGFR and anti-Notch (Millipore, USA, 1:500 dilution) were introduced and incubated with samples for 1 h, followed by additional 1 h of incubation the samples with goat anti-rabbit IgG-HRP (Abbkine, USA, 1:5000 dilution). Finally, the bands of interest were imaged and analyzed under the Fluor Chem Imaging System (Alpha Innotech, USA).

### Statistical analysis

All the results were displayed as mean ± standard deviation (SD) and the data were analyzed through unpaired Student's t-test and nonlinear regression. The level of statistical significance was set at *p* < 0.05 as it was considered to indicate a statistically significant difference.

## Results

### Characterization of nanoparticles

**Figure 1.**
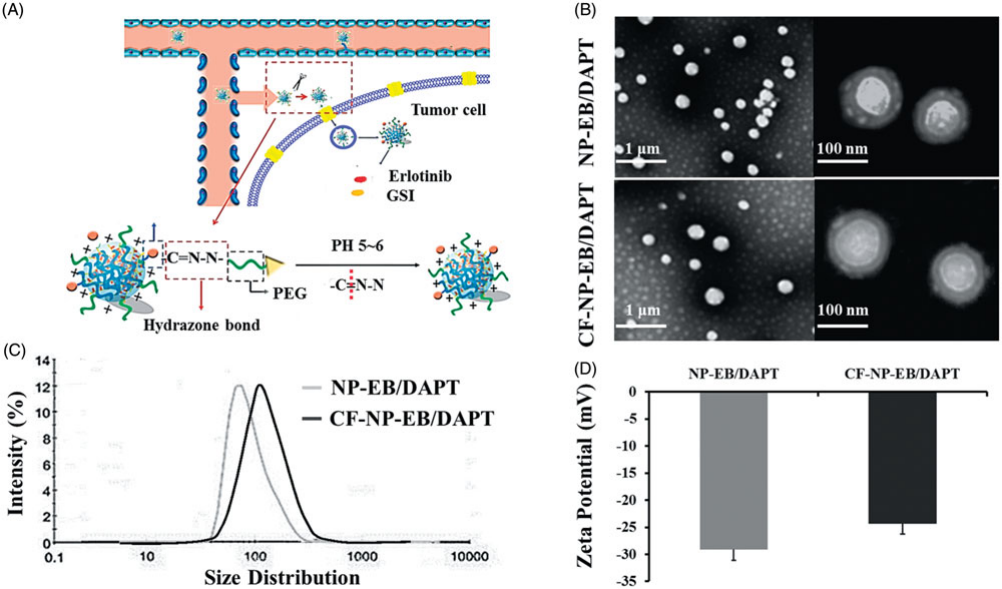
Characterization of the physicochemical properties of nanoparticles. (A) Schematic route of targeting delivery of drugs (EB and DAPT) to tumor tissues via mediating of CF peptide functionalized nanoparticles. (B) Morphologic images of NP-EB/DAPT and CF-NP-EB/DAPT obtained under the TEM. (C) Particle size distribution of NP-EB/DAPT and CF-NP-EB/DAPT determined by the DLS. (D) Zeta potential of NP-EB/DAPT and the unmodified nanoparticles.

The TEM images (Figure 1(B)) of the nanoparticles demonstrated that both the prepared NP-EB/DAPT and CF-NP-EB/DAPT were of vesicle-like shape with averaged diameters of 100 nm around, which is in good accordance with DLS results (96.37 ± 4.48 nm for NP-EB/DAPT and 104.65 ± 5.14 nm for the peptide modified ones). More importantly, as shown in Figure 1(C), both of nanoparticles displayed a narrow particle size distribution with the PDI values were 0.153 ± 0.054 (NP-EB/DAPT) and 0.179 ± 0.061 (CF-NP-EB/DAPT), respectively. We further investigate the zeta potential of nanoparticles and results revealed that values of NP-EB/DAPT and CF-NP-EB/DAPT were −29.1 ± 2.94 mV and −24.3 ± 3.13 mV, respectively, indicating that peptide conjugation had no influence on the zeta potential value of nanoparticles (Figure 1(D)). The successful conjugation of peptides on the surface of nanoparticles was confirmed by the XPS assay with results showed that the surface nitrogen detected on CF-NP-EB/DAPT was 0.56% while that on the surface of unconjugated NPs was undetectable. Moreover, replacing the drugs within nanoparticles with fluorescent dyes, such as the coumarin-6 or DiR, did not change the physicochemical properties of nanoparticles, such as the particle size, size distribution, and zeta-potential (Table 1).

**Table 1.** Characterization of the NP-EB/DAPT, CF-NP-EB/DAPT, CF-NP-C6, and CF-NP-DiR.

Nanoparticles	Particles Size(d.nm)	Polydispersityindex(PDI)	Zeta Potential(mV)
NP-EB/DAPT	96.37 ± 4.48	0.153 ± 0.054	−29.1 ± 2.94 mV
CF-NP-EB/DAPT	104.65 ± 5.14	0.179 ± 0.061	−24.3 ± 3.13 mV
CF-NP-C6	102.19 ± 3.81	0.143 ± 0.092	−27.4 ± 2.35 mV
CF-NP-DiR	107.28 ± 2.93	0.191 ± 0.039	−25.6 ± 3.82 mV

The drug loading capacity and entrapment efficiency of EB and DAPT in nanoparticles were investigated respectively. As results exhibited that the LC of EB in NP-EB/DAPT and CF-NP-EB/DAPT were 0.81 ± 0.74% and 0.79 ± 0.82%, respectively, while the EE of EB in NP-EB/DAPT and CF-NP-EB/DAPT were 44.81 ± 4.17% and 42.39 ± 3.86%, respectively. In terms of the DAPT, the LC of drugs in NP-EB/DAPT and CF-NP-EB/DAPT were 0.76 ± 0.58% and 0.72 ± 0.74%, respectively, while the EE of DAPT in NP-EB/DAPT and CF-NP-EB/DAPT were 40.25 ± 3.94% and 38.46 ± 4.21%, respectively. The results above suggested that the modification of CF peptide has negligible effect on the properties of nanoparticles and there was no significant difference between the drug loading efficacy of EB and DAPT.

Results of stability investigation exhibited that both NP-EB/DAPT and CF-NP-EB/DAPT did not show significant sizes increase during the experimental periods (Figure S1). These results suggested that the nanoparticles developed here possess a relative satisfactory stability for drug delivery.

### *In vitro* drug release studies

**Figure 2.**
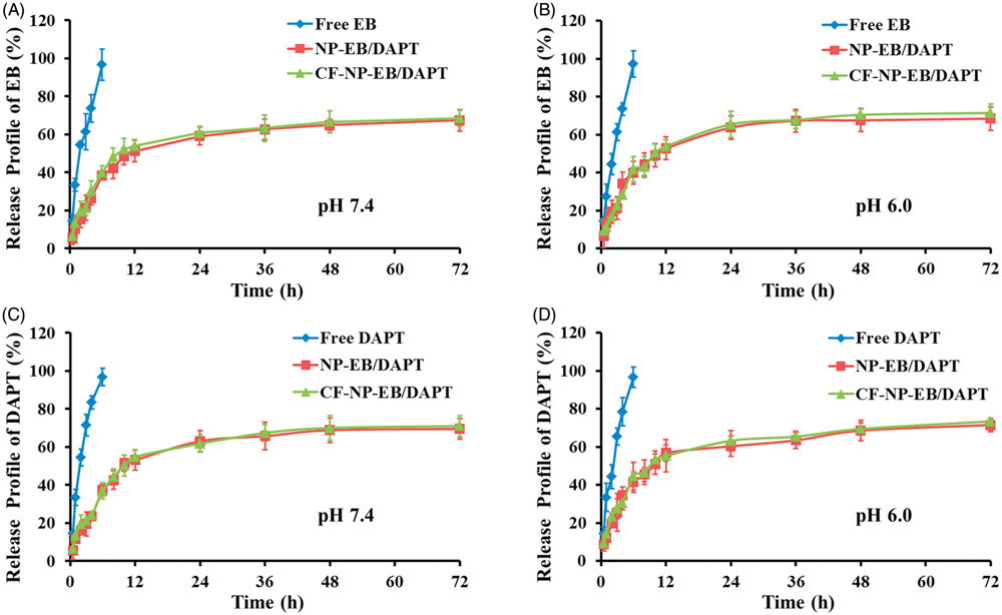
In vitro studies of the release behaviors of EB and DAPT from nanoparticles under different pH conditions. Release pattern of EB from NP-EB/DAPT and CF-NP-EB/DAPT under the conditions of pH 7.4 (A) and pH 6.0 (B). Release pattern of DAPT from NP-EB/DAPT and CF-NP-EB/DAPT under the conditions of pH 7.4 (C) and pH 6.0 (D).

Release behaviors of EB and DAPT from nanoparticles were performed, respectively, under different pH conditions. As shown in Figure 2(A,B), both the release profiles of EB from NP-EB/DAPT and CF-NP-EB/DAPT displayed a controlled release behavior, achieving cumulative drug release 68.49 ± 7.46% and 67.18 ± 5.21%, respectively, after 72 h incubation in PBS (pH 7.4). A similar release trend of DAPT from nanoparticles could be observed in Figure 2(C,D), with the cumulative drug release of 68.26 ± 6.37% and 70.45 ± 5.74%, respectively, after 72 h incubation in PBS (pH 7.4). These results indicated that the decoration of peptides has no effect on the drug release pattern and there were almost same release behaviors for EB and DAPT from nanoparticles. For comparison, free drugs including EB and DAPT were also subjected to the release evaluation. As results showed that both of EB and DAPT exhibited an initial burst release behavior and more than 96% free drugs were released within 6 h.

The peptide F3 and CREKA were conjugated with each other *via* a pH sensitive hydrazone bond, so we investigated whether the acidic tumor microenvironment significantly affect the release behaviors of EB and DAPT from nanoparticles. The results demonstrated that both the release profiles of EB and DAPT from different nanoparticles under the condition of pH 6.0 displayed a negligible difference from that of physiologic pH, suggesting that the acidic tumor microenvironment did not affect the drug release pattern of NP-EB/DAPT and CF-NP-EB/DAPT.

### Cellular uptake of nanoparticles

**Figure 3.**
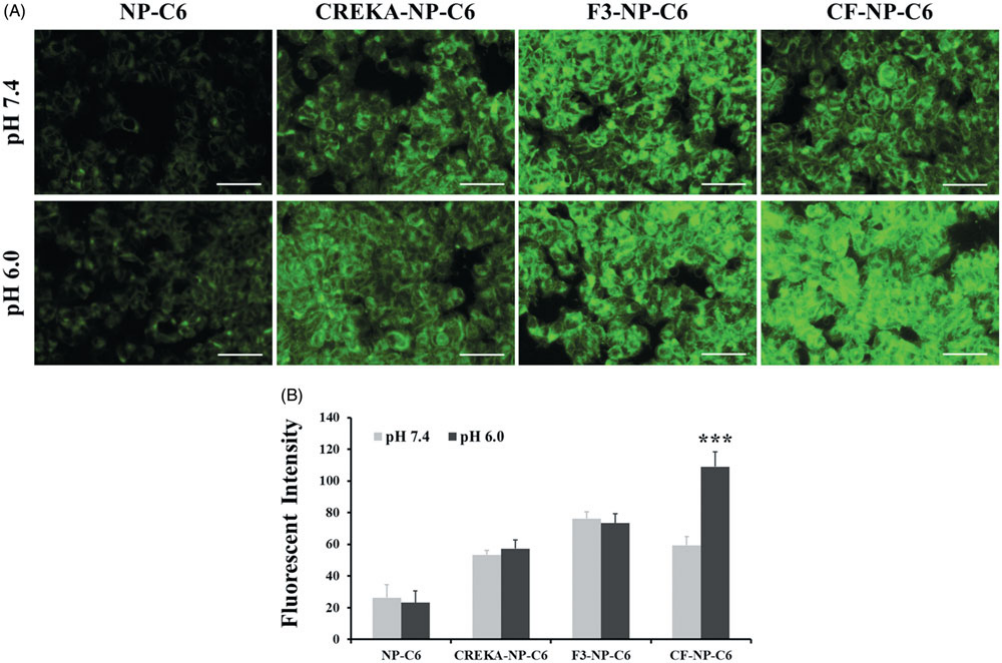
Evaluation of cellular uptake of nanoparticles in breast cancer cells. (A) Qualitative images of MDA-MB-231 cells after treatment of NP-C6, CREKA-NP-C6, F3-NP-C6, and CF-NP-C6, respectively, under different pH condition (pH 7.4 and pH 6.0). The bar represents 50 µm. (B) Quantitative analysis of cellular uptake of nanoparticles in MDA-MB-231 cells post treatment of various nanoparticle formulations.

Figure 3(A) displayed that MDA-MB-231 cells treated with NP-C6 displayed the weakest fluorescent intensity under both pH conditions. However, the fluorescent intensity in cancer cells was significantly elevated after incubated with peptide functionalized nanoparticles. Interestingly, under the physiological pH condition, MDA-MB-231 cells treated by CREKA-NP-C6 and CF-NP-C6 displayed a similar fluorescent intensity while the cells treated by F3-NP-C6 exhibited the strongest signal. Such result was mainly ascribed to the fact that the structure of CF peptide remains stable under the normal physical condition so that the cell penetrating capacity of F3 peptide was sealed. For verification, the experiments were also performed under the acidic environment with the cell culture medium was adjusted to pH 6.0. As results shown that the cells treated by CF-NP-C6 exhibited the strongest fluorescent signal when compared to other groups, indicating that the structure of CF peptide was disrupted under the acidic condition and cell penetrating capacity of F3 peptide was recovered. The above conclusions were further confirmed by the results of quantitative analysis obtained by the FCM evaluation (Figure 3(B)).

**Figure 4.**
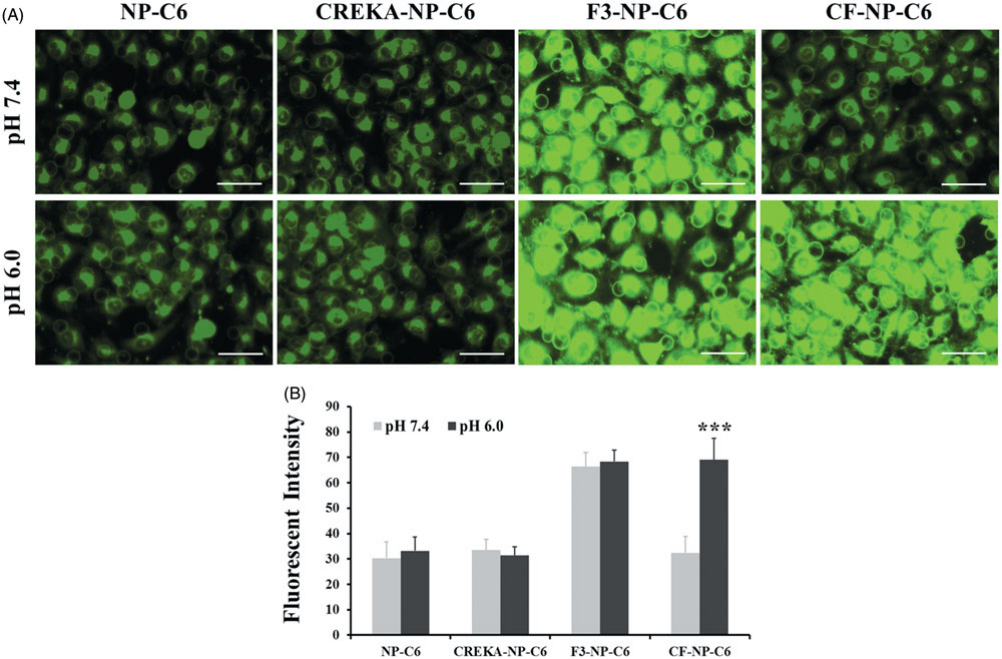
Evaluation of cellular uptake of nanoparticles in tumor angiogenesis. (A) Qualitative images of HUVEC cells after treatment of NP-C6, CREKA-NP-C6, F3-NP-C6, and CF-NP-C6, respectively, under different pH condition (pH 7.4 and pH 6.0). The bar represents 50 µm. (B) Quantitative analysis of cellular uptake of nanoparticles in HUVEC cells post treatment of various nanoparticle formulations. ***p < .001 significantly different with that of CF-NP-C6 (pH 7.4) group.

Moreover, the cellular uptake assay was also performed on the HUVEC cells to evaluate the affinity of nanoparticles to tumor angiogenesis. Results shown in Figure 4 demonstrated a similar cellular uptake behavior to that of breast cancer cells with the HUVEC cells treated by CF-NP-C6 (pH 6.0) achieving the strongest fluorescent intensity.

### Cytotoxicity of nanoparticles to tumor cells

**Figure 5.**
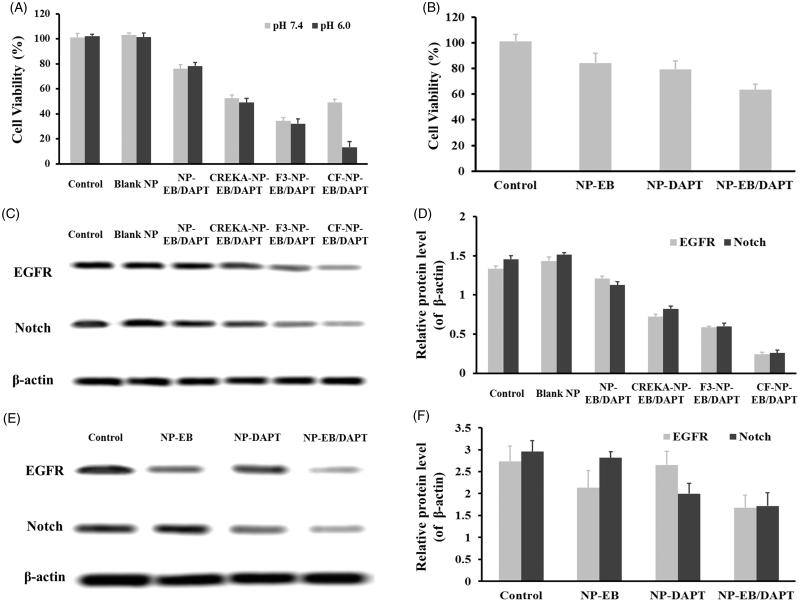
Cytotoxicity of different nanoparticles to tumor cells and effect of nanoparticles on the regulation of gene expressions. (A) Cell viability of MDA-MB-231 cells that treated by NP-EB/DAPT, CREKA-NP-EB/DAPT, F3-NP-EB/DAPT, and CF-NP-EB/DAPT, with drug concentrations ranging from 0.001 to 1 µg/mL. (B) Cell viability of MDA-MB-231 cells that was received the treatment of NP-EB, NP-DAPT, and NP-EB/DAPT. (C) Quantitative analysis and (D) qualitative evaluation of the levels of EGFR and Notch in tumor cells treated by NP-EB/DAPT, CREKA-NP-EB/DAPT, F3-NP-EB/DAPT, and CF-NP-EB/DAPT, respectively. (E) Quantitative analysis and (F) qualitative evaluation of the levels of EGFR and Notch in tumor cells treated by NP-EB, NP- DAPT, and NP-EB/DAPT, respectively.

Cytotoxicity of various nanoparticle formulations to breast cancer cells was evaluated by the MTT experiment. Cell viability columns shown in Figure 5(A) demonstrated an intensification of cell inhibition of CF-NP-EB/DAPT compared to NP-EB/DAPT under both pH conditions. Such results suggested a significantly improved cytotoxicity mediated by CF peptide, which was mainly due to the higher intracellular delivery CF-NP-EB/DAPT than that of unmodified ones. Moreover, in agreement with the cell uptake as presented above, cells treated by CF-NP-EB/DAPT and CREKA-NP-EB/DAPT exhibited the similar absorbancy under the pH 7.4 condition. However, the penetrating capacity of CF was recovered under the acidic environment with the cells incubated in pH 6.0 exhibited the lowest cell viability after treated by CF-NP-EB/DAPT. Of great importance, non-toxicity of blank NPs, which was unloaded with any drugs, to cells indicating that the developed nano-carriers are safety enough for drug delivery. Furthermore, the expression of EGFR and Notch in MDA-MB-231 cells post various treatments under pH 6.0 were further determined by western blot assay. As shown in Figure 5(C,D), after treatment of CF-NP-EB/DAPT, the levels of EGFR and Notch in tumor cells were obviously down-regulated while not for those cells treated by NP-EB/DAPT, indicating a good EGFR and Notch signaling inhibition effect for the peptides-modified nanoparticles.

The combination therapy effect of EB and DAPT was also performed under pH 6.0 using the same method as above except that the cells were treated by NP-EB, NP-DAPT, and NP-EB/DAPT, respectively. Cell viability columns shown in Figure 5(B) exhibited that proliferation of MDA-MB-231 cells could be significantly inhibited by treating with the EB and DAPT dual loaded nanoparticles. While the cells treated by NP-EB and NP-DAPT displayed a similar cell growth trend and both have higher cell viability than the cells treated by NP-EB/DAPT. The effect of these nanoparticles on regulating the expressions of EGFR and Notch was further determined. As shown in Figure 5(E,F), levels of EGFR and Notch in the cells treated by NP-EB/DAPT were down-regulated simultaneously, while the cells treated by NP-EB only displayed a decrease of EGFR expression and the cells treated by NP-DAPT only displayed a decrease of Notch expression. These results together indicated that the combination therapy of EB and DAPT could achieve a superior effect than the monotherapy of EB or DAPT.

### Inhibition of migration ability

**Figure 6.**
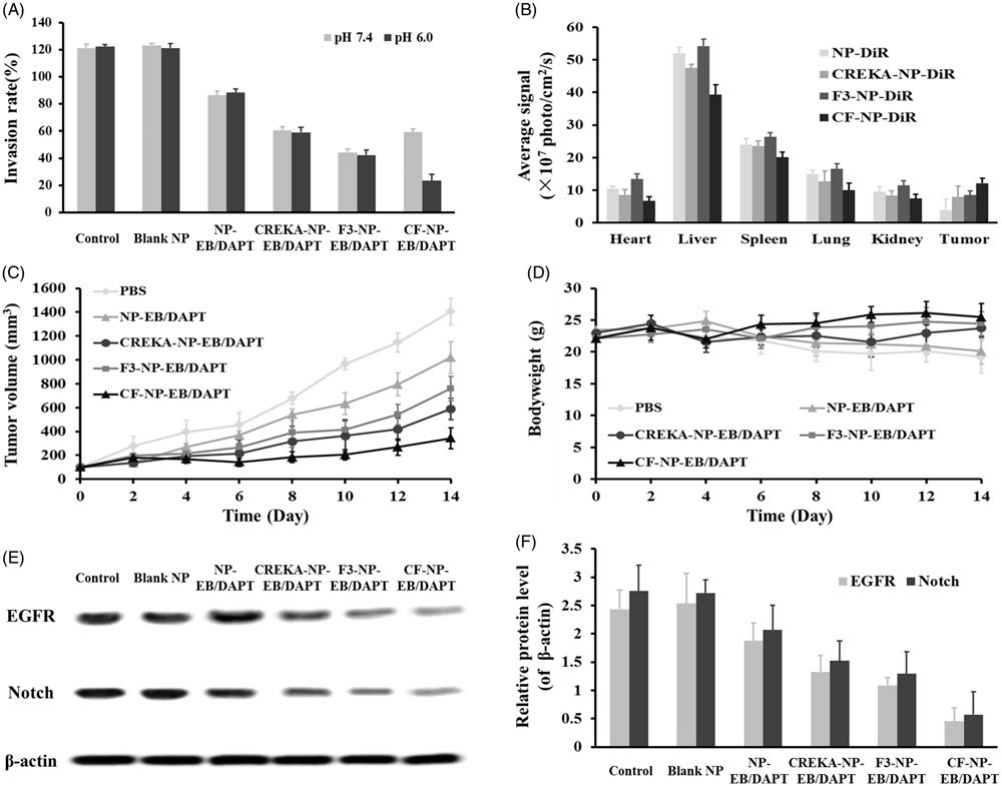
Evaluation of the in vivo tumor targeting efficacy and anti-tumor effect. (A) Tumor cell migration inhibition of various nanoparticle formulations. (B) Biodistribution of NP-DiR and CF-NP-DiR in tumor-bearing mice. (C) Changes of tumor volumes post treating tumor-bearing mice with different nanoparticles. (D) Body weight changes of tumor-bearing mice after treating by various nanoparticle formulations. (E) Levels of EGFR and Notch in tumor tissues after treatment of different and nanoparticles. (F) Quantitative analysis of the expressions of EGFR and Notch in tumor tissues.

Since the motility of tumor cells has been considered as an efficient measurement for evaluation of the metastatic potential of cancer cells, the transwell assay which focused on the vertical mobility was therefore investigated here. As shown in Figure 6(A), the cellular migration rates of the cells without any treatment or treated by blank NP was above 100%. In contrast, the NP-EB/DAPT displayed an obvious cell migration inhibition effect, with migration rate of 80%. Moreover, the migration inhibition effect of NP-EB/DAPT was further enhanced after decorating on the surface of nanoparticles with peptides. Of great importance, the cell penetrating ability of CF peptide was sealed under the normal physiological condition since tumor cells treated by CREKA-NP-EB/DAPT and CF-NP-EB/DAPT exhibited similar migration abilities. However, the migration inhibition effect of CF-NP-EB/DAPT was significantly enhanced when exposed tumor cells to acidic environment.

### Distribution of nanoparticles *in vivo*

To investigate the distribution of nanoparticles *in vivo*, tumor-bearing mice were respectively treated by DiR-labeled NP and CF-NP. Then the tumor sites and main organs were collected for nanoparticles detection. As shown in Figure 6(B), mice injected with peptides functionalized nanoparticles displayed a more accumulation at tumor sites when compared to the unmodified NPs. For the mice administrated with F3-NP, not only the tumor tissues exhibited higher fluorescent intensity compared to the control group, but also the normal organs. It was mainly due to the non-selectivity of F3 peptide. To address such issue, the penetrating capacity of F3 peptide was sealed by conjugation with CREKA *via* a pH-sensitive hydrazone bond. As results confirmed that mice treated by CF-NP displayed the lowest distribution in normal tissues especially the liver, indicating that the CF modified nanoparticles possess the best safety compared to other groups. However, when the CF-NP reached tumor sites, the hydrazone bond was disrupted in the acidic tumor environment and the cell penetrating capacity of F3 peptide was subsequently recovered. It was demonstrated by the fact that mice injected with CF-NP displayed the most amount of nanoparticle in tumor sites.

### Anti-tumor effect

After the tumor-bearing mice was received the treatment of different nanoparticle formulations, the tumor volumes were carefully observed and recorded. As shown in Figure 6(C), the growth of tumors in the mice treated by PBS was the fastest and could be significantly inhibited by treating with NP-EB/DAPT. Moreover, the tumor inhibition of NP-EB/DAPT was further enhanced by decorating on the surface of nanoparticles with CREKA and F3. Of great importance, the mice injected with CF-NP-EB/DAPT displayed the strongest tumor growth inhibition. Besides, the body weight of mice treated with saline, free drug and NP-EB/DAPT exhibited a downward trend. In contrast, a rising trend was observed in the mice treated with CREKA-NP-EB/DAPT, F-NP-EB/DAPT, and CF-NP-EB/DAPT (Figure 6(D)).

Furthermore, we investigated the effect of various nanoparticles on the expressions of EGFR and Notch in tumor tissues. As shown in Figure 6(E,F), the gene expressions were not affected by the treatment of blank NP, indicating the developed nano-carrier was non-toxicity. However, after co-loading with EB and DAPT, the effect of nanoparticles on inhibition the expressions of EGFR and Notch was significantly enhanced. Moreover, the peptides functionalized nanoparticles were obviously superior to the undecorated ones with the CF-NP-EB/DAPT exhibited the strongest inhibition ability of gene expressions.

## Discussion

Among the various forms of breast cancer, TNBCs represents the most aggressive ones and studies showed that only a small part of patients with TNBC can benefit from the current applied anti-cancer drugs (Pitner et al., 2017; Shi et al., 2017). For combating the growth of TNBCs, selecting appropriate related receptor is significantly critical. As the translational clinical medicine research displayed that high expression of EGFR is closely related to the malignant degree of breast cancer and leading to metastasis and poor prognosis (Brinkman et al., 2016). Additionally, the expression of EGFR in TNBCs was also critical to the development of strong chemotherapeutic resistance through maintaining intracellular glucose levels and protecting tumor cells from autophagy (Giró-Perafita et al., 2016). In this case, the EGFR has been selected as the standard target and EGFR-based therapy strategy has been widely accepted in treatment of TNBCs.

Unfortunately, previous reports pointed out that the benefits of EGFR-based therapy strategy could be signally impaired by the activated Notch signaling pathway *via* inducing resistance to EGFR inhibition (Chen & Russo, 2009). The activation of Notch signaling in cancer cells post erlotinib treatment finally resulted in an enriched stem cell-like population in a Notch and promoted the progress of cancers (Nowacka-Zawisza & Krajewska, 2013). Therefore, in the present study, we speculated that combined Notch-EGFR pathway inhibition might be a rational treatment strategy for the TNBCs. To achieve that goal, we developed the PLA-based nano-platforms and used the EGFR inhibitor EB and Notch inhibitor GSI-DAPT as model drugs. Cytotoxicity of NP-EB, NP-DAPT, and NP-EB/DAPT to MDA-MB-231 cells demonstrated that the combination treatment strategy holds great potential in combating with the growth of breast cancer, with cells treated by NP-EB/DAPT exhibited the lowest cell viability compared to cells received monotherapy.

A wide array of physicochemical properties such as the particle size, zeta-potential, and surface morphology significantly affect the pharmacokinetics (PK) and biodistribution of nanoparticles-based drug delivery systems (Alexis et al., 2008; Kulkarni & Feng, 2013; Liu et al., 2018a). It was widely accepted that around 100 nm is a relative ideal particle size because it is optimal for taking full advantage of enhanced permeability and retention (EPR) effect (Kulkarni & Feng, 2013). Herein, the developed NP-EB/DAPT and various peptide-modified ones were at a mean diameter of 100 nm around, which are suitable for improving pharmacokinetics and biodistribution. Furthermore, all nanoparticles were developed by highly hydrophilic PEG content polymers, indicating a good resistance to opsonization and a good stability by decreasing MPS uptake and prolonging blood circulation (Kulkarni & Feng, 2013).

Similar release pattern was observed for both NP-EB/DAPT and CF-NP-EB/DAPT, suggesting that the modification of CF peptides on the nanoparticulate surface did not change the release behavior of drugs. Although the CF peptide has been designed to be acid-cleavable, no significant difference was detected in the release medium of pH 6.0 and pH 7.4. This is reasonable because the modification of peptide was realized by coupling the CF molecules to the carboxyl groups which were located on the surface of nanoparticles while not the polymer matrix. More importantly, both of EB and DAPT displayed a biphasic pattern which is characterized by an initial fast release and a following controlled release.

Efficiently treatment of solid cancer is dramatically restricted by the non-selectivity of anti-tumor drugs and insufficient tumor accumulation or inner penetration of chemotherapeutics (Feng et al., 2017; Gao, 2016; Liu et al., 2018b). For enhancing the tumor selectivity of drugs, we decorated on the surface of nanoparticles with CREKA peptide, a linear pentapeptide was designed to bind to fibrous protein which are abundant in various types of tumors but not in normal tissues (Simberg et al., 2007; Song et al., 2014). The modification resulted in a higher accumulation of nanoparticles in MDA-MB-231 cells and tumor tissues. To further improve the tumor inner penetration of nanoparticles, the namely cell penetrating peptide F3 was further decorated on the surface of drugs-loaded nanoparticles. However, the modification of F3 peptide not only enhanced the accumulation of nanoparticles at tumor sites, but also the normal tissues, which will resulted in serious side effect. Cumulative studies have demonstrated that the unique tumor environment, especially the acidic pH, could be employed to design various smart systems for site specific drug delivery (Hu et al., 2018; Yang et al., 2017). In this case, the F3 peptide was conjugated with CREKA through the acidic-cleavable hydrazone bond to build a newly peptide, CF. By this way, the cell penetrating ability of F3 peptide was sealed under normal condition and will be recovered when reached tumor sites.

In our studies, cancer cells cultured in normal physiological condition treated by coumarin-6 labeled-CREKA-NP and CF-NP exhibited similar fluorescent intensity, indicated that the structure of CF was remain intact. Under the normal physiological condition (pH 7.4), cells treated by coumarin-6 labeled-CREKA-NP and CF-NP exhibited similar fluorescent intensity, indicated that the structure of CF remained intact. It can be confirmed by further evaluation under the acidic environment (pH 6.0) with cells treated by CF-NP displayed the strongest green signal due to the uncovered cell penetration ability of F3 peptide. Consistently, sealing the cell penetrating capacity of F3 peptide leaded to a dramatically decreased cytotoxicity of CF-NP-EB/DAPT to cells. However, the cytotoxicity of CF-NP-EB/DAPT to cells could be rapidly enhanced under acidic culture medium. Furthermore, the expression of EGFR and Notch in MDA-MB-231 cells were obviously down-regulated after treatment of CF-NP-EB/DAPT while not for those cells treated by NP-EB/DAPT, indicating a well EGFR and Notch signaling inhibition effect for the peptides-modified nanoparticles.

Furthermore, the mice received the treatment of CF-NP-DiR showed the maximum amount of nanoparticles accumulation, indicating the structure of CF could be tremendously disrupted under the acidic tumor environment. The excellent tumor targeting ability and triggered penetration of CF peptide at the specific tumor site resulted in an anticipated tumor progress inhibition. Compared with the NP-EB/DAPT, CREKA-NP-EB/DAPT or F3-NP-EB/DAPT, CF peptide-modified nanoparticles exhibited the strongest antitumor activity with the tumor volumes were significantly decreased.

## Conclusion

In the present study, we have developed PLA-based nano-platforms and used erlotinib (EB) as a model drug to treat TNBCs. Recognizing the significant role of Notch-EGFR interplay in raising severe resistance to EGFR inhibition of EB, gamma secretase inhibitor (GSI)-DAPT was further introduced to inhibit the activation of Notch signaling. For the purpose of enhancing accumulation of drugs at tumor site and achieving a desirable tumor inner penetration, a tumor homing peptide CREKA and cell penetrating F3 peptide were decorated on the surface of EB and DAPT-dual loaded nanoparticles (CF-NP-EB/DAPT). The tumor inhibition efficacy of CF-NP-EB/DAPT has been well confirmed by a series of experiments such as in vitro cellular uptake assay, cytotoxicity evaluation, in vivo tumor targeting study and anti-tumor effect investigation. In a word, the prepared EGFR pathway and Notch signaling-dual recognizable nanoparticles might represent a promising strategy for enhancing therapy efficacy of TNBCs.

## Supplementary Material

Supplementary_Materials.docx

## References

[CIT0001] Alexis F, Pridgen E, Molnar LK, et al. (2008). Factors affecting the clearance and biodistribution of polymeric nanoparticles. Mol Pharm 5:505–15. 18672949 10.1021/mp800051m PMC2663893

[CIT0002] Andersson ER, Sandberg R, Lendahl U (2011). Notch signaling: simplicity in design, versatility in function. Development 138:3593–612. 21828089 10.1242/dev.063610

[CIT0003] Arasada RR, Amann JM, Rahman MA, et al. (2014). EGFR blockade enriches for lung cancer stem-like cells through Notch3-dependent signaling. Cancer Res 74:5572–84. 25125655 10.1158/0008-5472.CAN-13-3724 PMC4263272

[CIT0004] Baker AT, Zlobin A, Osipo C (2014). Notch-EGFR/HER2 bidirectional crosstalk in breast cancer. Front Oncol 4:360. 25566499 10.3389/fonc.2014.00360 PMC4264417

[CIT0005] Baselga J, Arteaga CL (2005). Critical update and emerging trends in epidermal growth factor receptor targeting in cancer. J Clin Oncol 23:2445–59. 15753456 10.1200/JCO.2005.11.890

[CIT0006] Bauer KR, Brown M, Cress RD, et al. (2007). Descriptive analysis of estrogen receptor (ER)-negative, progesterone receptor (PR)-negative, and HER2-negativeinvasive breast cancer, the so-called triple-negative phenotype: a population-based study from the California cancer Registry. Cancer 109:1721–8. 17387718 10.1002/cncr.22618

[CIT0007] Brinkman AM, Chen G, Wang Y, et al. (2016). Aminoflavone-loaded EGFR-targeted unimolecular micelle nanoparticles exhibit anti-cancer effects in triple negative breast cancer. Biomaterials 101:20–31. 27267625 10.1016/j.biomaterials.2016.05.041 PMC5030715

[CIT0008] Chen JQ, Russo J (2009). ERalpha-negative and triple negative breast cancer: molecular features and potential therapeutic approaches. Biochim Biophys Acta 1796:162–75. 19527773 10.1016/j.bbcan.2009.06.003 PMC2937358

[CIT0009] Corkery B, Crown J, Clynes M, O'Donovan N (2009). Epidermal growth factor receptor as a potential therapeutic target in triple-negative breast cancer. Ann Oncol 20:862–7. 19150933 10.1093/annonc/mdn710

[CIT0010] Crown J, O'Shaughnessy J, Gullo G (2012). Emerging targeted therapies in triple-negative breast cancer. Ann Oncol 23:56–65. 23012305 10.1093/annonc/mds196

[CIT0011] Efferth T (2012). Signal transduction pathways of the epidermal growth factor receptor in colorectal cancer and their inhibition by small molecules. CMC 19:5735–44. 10.2174/092986712803988884 23033949

[CIT0012] Farnie G, Clarke RB (2007). Mammary stem cells and breast cancer-role of Notch signalling. Stem Cell Rev 3:169–75. 17873349 10.1007/s12015-007-0023-5

[CIT0013] Feng X, Yao J, Gao X, et al. (2015). Multi-targeting peptide-functionalized nanoparticles recognized vasculogenic mimicry, tumor neovasculature, and glioma cells for enhanced anti-glioma therapy. ACS Appl Mater Interfaces 7:27885–99. 26619329 10.1021/acsami.5b09934

[CIT4013] Feng X, Yao J, Gao X, et al. (2017). Multi-targeting peptide-functionalized nanoparticles recognized vasculogenic mimicry, tumor neovasculature, and glioma cells for enhanced anti-glioma therapy. ACS Appl Mater Interfaces 7:27885–99. 10.1021/acsami.5b09934 26619329

[CIT0014] Gao H (2016). Progress and perspectives on targeting nanoparticles for brain drug delivery. Acta Pharm Sin B 6:268–86. 27471668 10.1016/j.apsb.2016.05.013 PMC4951594

[CIT0015] Gao H (2017). Perspectives on dual targeting delivery systems for brain tumors. J Neuroimmune Pharmacol 12:6–16. 27270720 10.1007/s11481-016-9687-4

[CIT0016] Giró-Perafita A, Palomeras S, Lum DH, et al. (2016). Preclinical evaluation of fatty acid synthase and EGFR inhibition in triple-negative breast cancer. Clin Cancer Res 22:4687–97. 27106068 10.1158/1078-0432.CCR-15-3133

[CIT0017] Hu C, Cun X, Ruan S, et al. (2018). Enzyme-triggered size shrink and laser-enhanced NO release nanoparticles for deep tumor penetration and combination therapy. Biomaterials 168:64–75. 29626787 10.1016/j.biomaterials.2018.03.046

[CIT0018] Kulkarni SA, Feng SS (2013). Effects of particle size and surface modification on cellular uptake and biodistribution of polymeric nanoparticles for drug delivery. Pharm Res 30:2512–22. 23314933 10.1007/s11095-012-0958-3

[CIT0019] Li J, Chen L, Liu N, et al. (2016). EGF-coated nano-dendriplexes for tumor-targeted nucleic acid delivery in vivo. Drug Deliv 23:1718–25. 25693638 10.3109/10717544.2015.1004381

[CIT0020] Liao MJ, Ye MN, Zhou RJ, et al. (2014). Yiqi formula enhances the antitumor effects of erlotinib for treatment of triple-negative breast cancer xenografts. Evid Based Complement Alternat Med 2014:628712. 25389442 10.1155/2014/628712 PMC4217362

[CIT0021] Liu CY, Huang TT, Huang CT, et al. (2017). EGFR-independent Elk1/CIP2A signalling mediates apoptotic effect of an erlotinib derivative TD52 in triple-negative breast cancer cells. Eur J Cancer 72:112–23. 28027514 10.1016/j.ejca.2016.11.012

[CIT0023] Liu R, Xiao W, Hu C, et al. (2018a). Theranostic size-reducible and no donor conjugated gold nanocluster fabricated hyaluronic acid nanoparticle with optimal size for combinational treatment of breast cancer and lung metastasis. *J Control Release* 278:127–39. 29630985 10.1016/j.jconrel.2018.04.005

[CIT0022] Liu R, Hu C, Yang Y, et al. (2018b). Theranostic nanoparticles with tumor-specific enzyme-triggered size reduction and drug release to perform photothermal therapy for breast cancer treatment. *Acta Pharm Sin B Online*. 10.1016/j.apsb.2018.09.001 PMC6438824 30976492

[CIT0024] Masuda H, Zhang D, Bartholomeusz C, et al. (2012). Role of epidermal growth factor receptor in breast cancer. Breast Cancer Res Treat 136:331–45. 23073759 10.1007/s10549-012-2289-9 PMC3832208

[CIT0025] Nowacka-Zawisza M, Krajewska WM (2013). Triple-negative breast cancer: molecular characteristics and potential therapeutic approaches. Postepy Hig Med Dosw (Online) 67:1090–7. 24379250 10.5604/17322693.1077713

[CIT0026] Palma G, Frasci G, Chirico A, et al. (2015). Triple negative breast cancer: looking for the missing link between biology and treatments. Oncotarget 6:26560–74. 26387133 10.18632/oncotarget.5306 PMC4694936

[CIT0027] Pitner MK, Taliaferro JM, Dalby KN, et al. (2017). MELK: a potential novel therapeutic target for TNBC and other aggressive malignancies. *Expert Opin Ther Targets* 21:849–59. 28764577 10.1080/14728222.2017.1363183

[CIT0028] Porkka K, Laakkonen P, Hoffman JA, et al. (2002). A fragment of the HMGN2 protein homes to the nuclei of tumor cells and tumor endothelial cells in vivo. Proc Natl Acad Sci USA 99:7444–9. 12032302 10.1073/pnas.062189599 PMC124250

[CIT0029] Quan X, Gao H, Wang Z, et al. (2018). Epidermal growth factor receptor somatic mutation analysis in 354 Chinese patients with non-small cell lung cancer. Oncol Lett 15:2131–8. 29434916 10.3892/ol.2017.7622 PMC5776883

[CIT0030] Robinson DR, Kalyana-Sundaram S, Wu YM, et al. (2011). Functionally recurrent rearrangements of the MAST kinase and Notch gene families in breast cancer. Nat Med 17:1646–51. 22101766 10.1038/nm.2580 PMC3233654

[CIT0031] Ruan S, Xiao W, Hu C, et al. (2017). Ligand-mediated and enzyme-directed precise targeting and retention for the enhanced treatment of glioblastoma. ACS Appl Mater Interfaces 9:20348–60. 28557433 10.1021/acsami.7b02303

[CIT0032] Shi M, Ma F, Liu J, et al. (2017). A functional BRCA1 coding sequence genetic variant contributes to prognosis of triple-negative breast cancer, especially after radiotherapy. Breast Cancer Res Treat 166:109–16. 28744749 10.1007/s10549-017-4395-1

[CIT0033] Simberg D, Duza T, Park JH, et al. (2007). Biomimetic amplification of nanoparticle homing to tumors. Proc Natl Acad Sci USA 104:932–6. 17215365 10.1073/pnas.0610298104 PMC1783417

[CIT0034] Song Y, Huang Z, Xu J, et al. (2014). Multimodal SPION-CREKA peptide based agents for molecular imaging of microthrombus in a rat myocardial ischemia-reperfusion model. Biomaterials 35:2961–70. 24393265 10.1016/j.biomaterials.2013.12.038

[CIT0035] Xiao W, Ruan S, Yu W, et al. (2017). Normalizing tumor vessels to increase the enzyme-induced retention and targeting of gold nanoparticle for breast cancer imaging and treatment. Mol Pharm 14:3489–98. 28845990 10.1021/acs.molpharmaceut.7b00475

[CIT0036] Yan J, Wang Y, Jia Y, et al. (2017). Co-delivery of docetaxel and curcumin prodrug via dual-targeted nanoparticles with synergistic antitumor activity against prostate cancer. Biomed Pharmacother 88:374–83. 28122302 10.1016/j.biopha.2016.12.138

[CIT0037] Yang S, Gao H (2017). Nanoparticles for modulating tumor microenvironment to improve drug delivery and tumor therapy. Pharmacol Res 126:97–108. 28501517 10.1016/j.phrs.2017.05.004

