# Supplementary material for: pH sensitive peptide functionalized nanoparticles for co-delivery of erlotinib and DAPT to restrict the progress of triple negative breast cancer
Source: Drug Deliv. 2019 Apr 8;26(1):470–80. doi: 10.1080/10717544.2019.1576801 (PMC6462792; doi:10.1080/10717544.2019.1576801)
Supplement: Supplementary_Materials.docx [file IDRD_A_1576801_SM1027.docx]

**Supplementary Materials**


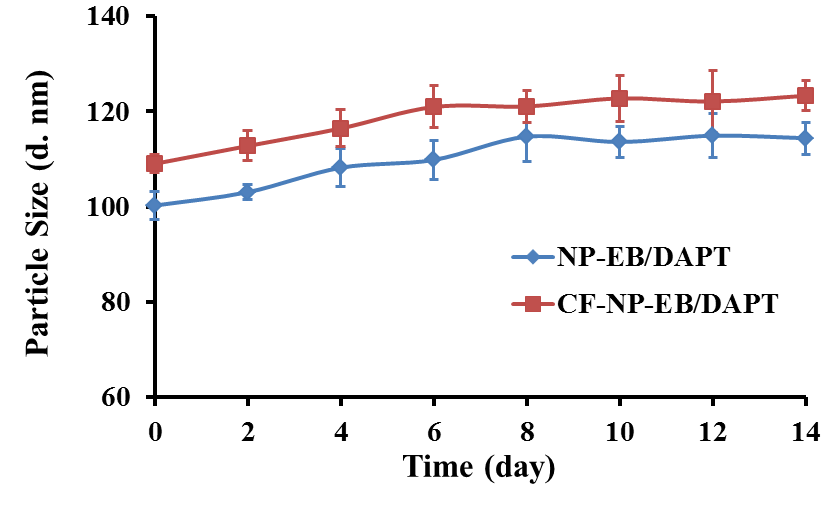


**Figure S1.** Stability investigation of the developed NP-EB/DAPT and CF-NP-EB/DAPT with the saline containing 10 % rat plasma acting as the medium.
